# Zebrafish are Resistant to *Staphylococcus aureus* Endophthalmitis

**DOI:** 10.3390/pathogens8040207

**Published:** 2019-10-26

**Authors:** Frank Mei, Matthew Rolain, Xiao Yi Zhou, Pawan Kumar Singh, Ryan Thummel, Ashok Kumar

**Affiliations:** 1 Wayne State University School of Medicine, Detroit, MI 48201, USA; fmei@med.wayne.edu (F.M.); mrolain@med.wayne.edu (M.R.);; 2 Department of Ophthalmology, Visual and Anatomical Sciences, Wayne State University School of Medicine, Detroit, MI 48201, USA; psing@med.wayne.edu; 3 Department of Biochemistry, Microbiology, and Immunology, Wayne State University School of Medicine, Detroit, MI 48201, USA

**Keywords:** *S. aureus*, zebrafish, host–pathogen interaction, eye, innate immunity

## Abstract

Gram-positive bacteria remain the leading cause of endophthalmitis, a blinding infectious disease of the eye. Murine models have been widely used for understanding the pathogenesis of bacterial endophthalmitis. In this study, we sought to develop an alternative zebrafish (*Danio rerio*) model for *Staphylococcus aureus* and compare the disease pathobiology to a murine model. Endophthalmitis was induced in zebrafish and C57BL/6 mice through the intravitreal injection of *S. aureus*. Disease progression was monitored by assessing corneal haze, opacity, bacterial burden, and retinal histology. Our results demonstrated that, unlike the murine models, zebrafish maintained ocular integrity, corneal transparency, and retinal architecture. We found that the zebrafish was capable of clearing *S. aureus* from the eye via transport through retinal vessels and the optic nerve and by mounting a monocyte/macrophage response beginning at 8 hour post-infection (hpi). The bacterial burden increased up to 8 hpi and significantly decreased thereafter. An assessment of the innate retinal response revealed the induced expression of *Il-1β* and *Il-6* transcripts. Collectively, our study shows that unlike the murine model, zebrafish do not develop endophthalmitis and rapidly clear the pathogen. Hence, a better understanding of the zebrafish protective ocular innate response may provide new insights into the pathobiology of bacterial endophthalmitis.

## 1. Introduction

Bacterial endophthalmitis is a devastating ocular infection, which if not diagnosed and treated quickly, can result in vision loss within a few hours [1]. Any ocular perforation (surgery and/or trauma) predisposes a patient to developing endophthalmitis. Cataract surgery, one of the most common surgical procedures performed among the aged population worldwide, has a higher incidence of endophthalmitis than any other type of ocular surgery [2,3]. The incidence of endophthalmitis has also steadily risen over the past two decades due to the increased popularity of suture-less cataract surgery, small-gauge vitrectomy, and intravitreal injections of anti-vascular endothelial growth factor (VEGF) drugs for treating age-related macular degeneration and diabetic macular edema [4,5].

Innate immunity provides the first line of defense against bacterial infections [6,7]. However, the eye, being an immune-privileged site, provides an immunosuppressive environment for the growth and proliferation of microbial pathogens [8]. Moreover, the eye is isolated from systemic circulation due to the presence of the blood–retinal barrier (BRB), making it difficult for the immune system to reach the site of infection [9]. Traditionally, murine models have been used for studying innate defense mechanisms and disease pathology against bacterial endophthalmitis [10,11,12]. However, the scientific community continues to investigate new models to reduce the cost and high demands of animals. Vertebrate fish models have been utilized as a model organism for several infectious diseases and innate immune system studies. Previous studies have demonstrated that the zebrafish (*Danio rerio*) model is a valuable model for the study of host–pathogen interactions and immune mechanisms against several bacterial infections, including *Streptococcus* [13], *Vibrio cholera* [14,15], and *Salmonella* [16,17]. The zebrafish has many similarities to the human immune system, and its well-developed genetics, small size, and rapid generation time has made it an easy choice as a model for several disease, developmental, and immune system studies [18,19,20,21,22,23,24]. However, the feasibility of zebrafish as an alternative model for endophthalmitis has never been explored. 

The aim of this study was to develop a zebrafish model of *Staphylococcus aureus* endophthalmitis and to compare the disease pathobiology to an established murine model. We report that in comparison to the murine model, zebrafish were able to rapidly clear the inoculated bacteria from the eyes and did not develop endophthalmitis.

## 2. Results

### 2.1. Zebrafish Did Not Develop Staphylococcal Endophthalmitis

**Figure 1:**
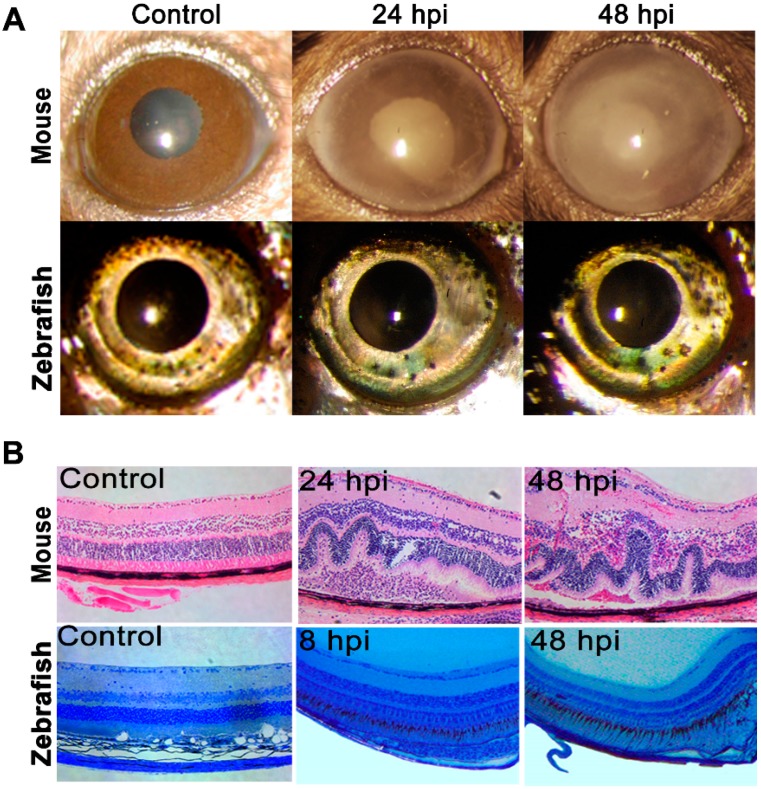
Endophthalmitis was induced through intravitreal injection of *Staphylococcus aureus* in wild type (WT) C57BL/6 mice (*n* = 6) eyes (5000 CFU/eye) and in zebrafish (*Danio rerio*, AB strain) (*n* = 10) eyes (250,000 CFU/eye). PBS-injected eyes were used as a control. (**A**) A slit-lamp microscopy examination was performed on the eyes of both mice and zebrafish, and micrographs were taken for representative eyes at indicated time points. (**B**) For a histological analysis, eyes were enucleated at indicated time points and subjected to hematoxylin and eosin (H & E) staining for mice and Methylene Blue-Azure II for zebrafish eyes.

Since zebrafish have never been evaluated for bacterial endophthalmitis, we sought to compare their susceptibility to an established mouse model of staphylococcal endophthalmitis [7,12,25,26]. The eyes of both B6 mice and a zebrafish (*Danio rerio*) AB strain were inoculated with 5000 colony-forming unit (CFU) of *S. aureus*. At 24 and 48 h post-infection, the mice eyes exhibited severe corneal haze, opacity, and hypopyon compared to the phosphate-buffered saline (PBS)-injected controls (Figure 1A, upper panel). Coinciding with this phenotype, the mice eyes also exhibited severe damage in the retinal architecture, including retinal folding, a loss of retinal demarcation, and heavy cellular infiltration (Figure 1B, upper panel). However, *S. aureus* at a 5000 CFU/eye dose failed to cause any ocular pathology in zebrafish (data not shown). Next, we gradually increased the infective dose of *S. aureus* and observed that even at 250,000 CFU/eye (i.e., a 50-fold higher dose compared to the mice), the zebrafish corneas appeared clear with no signs of inflammation when compared to uninfected eyes under slit-lamp examination (Figure 1A, lower panel). Furthermore, a histological analysis also revealed intact retinal architecture without any sign of retinal folding, cellular infiltrates, or damage (Figure 1B, lower panel). 

**Figure 2:**
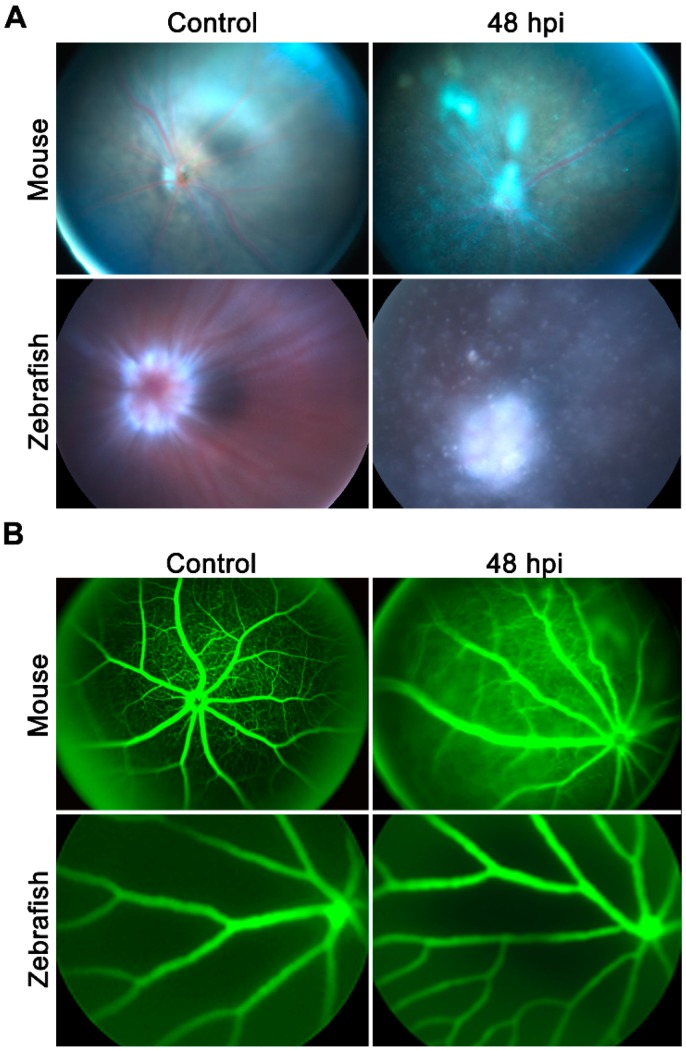
Endophthalmitis was induced by intravitreal injection of *S. aureus* in WT C57BL/6 mice (*n* = 6) eyes (5000 CFU/eye) and in zebrafish (*n* = 10) eyes (250,000 CFU/eye). PBS-injected eyes were used as a control. (**A**) A fundoscopic examination was performed on the eyes of both mice and zebrafish using Micron 3, and images were taken for representative eyes at indicated time points. (**B**) An angiography was performed through the injection of 2% fluorescent dye into the peritoneum of the mice and the caudal artery of the zebrafish using Micron 3.

Because infected eyes are known to have a breakdown of BRB causing increased vascular permeability [11], we compared both mice and zebrafish eyes using fundus imaging combined with angiography 48 h post-infection. Our results showed that *S. aureus* infection induced severe vitreous inflammation with opaque media along with vascular leakage (Figure 2A,B, upper panels) in mice eyes. In contrast, zebrafish showed comparatively reduced vitreous inflammation with no sign of vascular leakage in their fundus (Figure 2A,B, lower panels). These results indicate that despite a higher bacterial inoculation dose, the zebrafish were capable of protecting their eyes from *S. aureus* endophthalmitis.

### 2.2. Zebrafish Rapidly Cleared Bacterial Burden from Eyes

**Figure 3:**
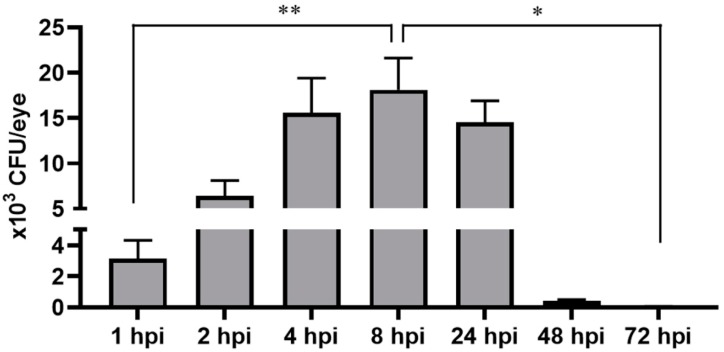
Zebrafish eyes (*n* = 10) were infected with *S. aureus* (250,000 CFU/eye). At indicated time points, eyes were enucleated and homogenized, and the bacterial burden was estimated via serial dilution plating (* *p* < 0.05; ** *p* < 0.005; Student’s *t*-test).

The study in the mice model showed a time-dependent increase in bacterial burden that coincided with increased retinal tissue damage. Since we did not observe an ocular pathology in *S. aureus*-infected zebrafish eyes, we postulated that the intraocular milieu of the zebrafish eye is not conducive for bacterial survival. To examine this possibility, we estimated the overall bacterial burden at various time points following *S. aureus* inoculation. Our data showed a time-dependent increase in bacterial burden up to 8 hpi followed by a drastic decline at 48 and 72 hpi (Figure 3).

### 2.3. Zebrafish Eyes Cleared S. aureus through the Vasculature and Optic Nerve Head

**Figure 4:**
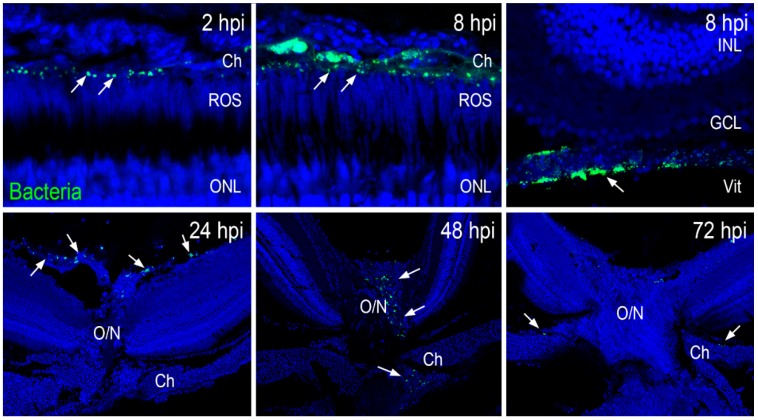
Zebrafish eyes (*n* = 10) were infected with GFP-positive *S. aureus* (250,000 CFU/eye). At indicated time points, retinal cryosections were immunostained for GFP (green) and colabeled with TO-PRO 3 for nuclei (blue). *S. aureus* (shown by white arrows) was initially seen in choroidal and vitreous vasculature, with the remaining bacteria being cleared through the optic nerve head with time. INL: inner nuclear layer; GCL: ganglion cell layer; Vit: vitreous chamber; O/N: optic nerve; Ch: choroid; ROS: rod outer segment.

Given the clearance of bacteria in the zebrafish eyes, we used immunohistochemistry to visualize the distribution of green fluorescent protein (GFP)-positive *S. aureus* in retinal sections at various time points postinfection. Our data showed that at 2 and 8 hpi, GFP-positive *S. aureus* (green) was localized in the choroidal and vitreous vasculature (Figure 4). Interestingly, at 8 hpi, a large amount of *S. aureus* seemed to colocalize with blood cells adhering to retinal blood vessels. At 24 hpi, many *S. aureus* were located in a blood vessel near the optic nerve head, and at 48 hpi, *S. aureus* was visualized passing through the optic nerve head. Finally, at 72 hpi, there were fewer *S. aureus* traversing through the optic nerve (Figure 4). At this time point, no *S. aureus* were observed in the retina, which was consistent with the preservation of retinal architecture at 24 and 48 hpi. These results indicate that zebrafish eliminated bacteria from their eyes via retinal blood vessels and traversal through the optic nerve. 

### 2.4. Zebrafish Eyes Recruited Monocytes/Macrophages during S. aureus Infection

**Figure 5:**
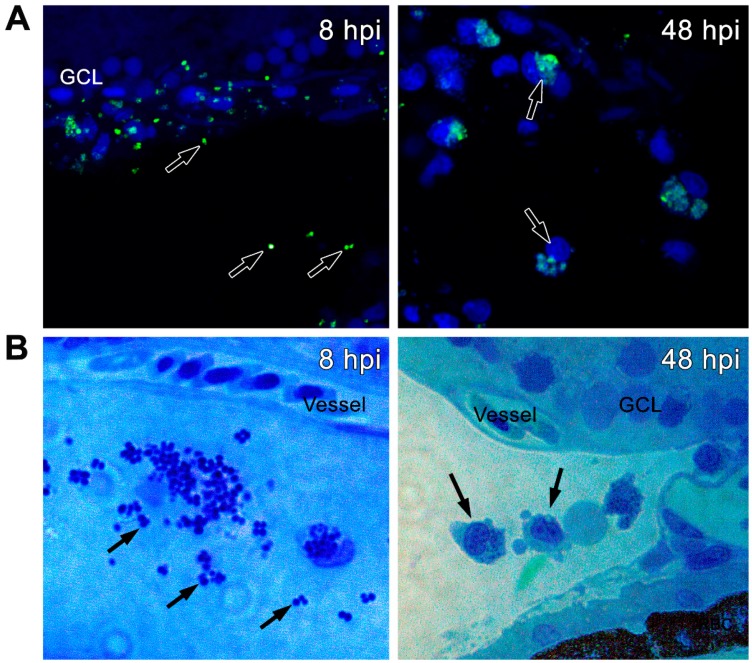
Zebrafish eyes (*n* = 10) were infected with GFP-positive *S. aureus* (250,000 CFU/eye). (**A**) Retinal cryosections were immunostained with anti-GFP antibody (green) and colabeled with TO-PRO 3 for nuclei (blue). *S. aureus* (as shown by black arrows) was located in the vitreous as well as in phagocytic cells. (**B**) Retinal sections were stained with Tetrazolium blue at indicated time points. The stained cells appeared to be macrophages with engulfed *S. aureus* in the vitreous cavity.

Bacteria observed in the choroidal and vitreous vasculature appeared to be largely free-floating and not contained within a phagocytic cell. Outside of the vasculature, we observed large numbers of free-floating *S. aureus* in the vitreous immediately adjacent to the ganglion cell layer and vitreous vasculature at 8 and 48 hpi, including some inside of phagocyte-appearing cells (Figure 5A). To characterize these cells, we used Methylene Blue-Azure II staining. At 8 hpi as well as at 48 hpi, we observed bacteria in the limbal region of the vitreous within cells with a round nucleus and pseudopods, which are characteristic of monocytes/macrophages (Figure 5B). These data indicated that the phagocytic actions of infiltrated macrophages/monocytes mediated the *S. aureus* clearance in zebrafish eyes.

### 2.5. Infected Zebrafish Eyes Differentially Expressed Inflammatory Mediators

**Figure 6:**
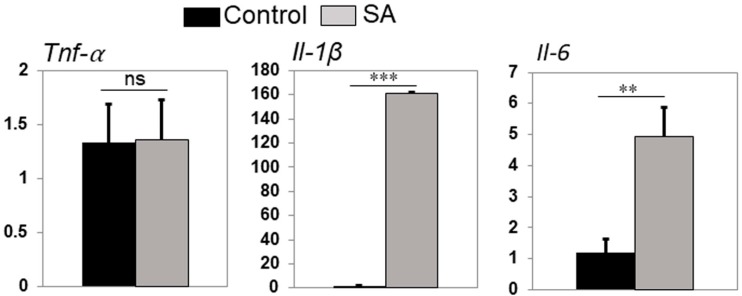
Zebrafish eyes (*n* = 10) were infected with *S. aureus* (250,000 CFU/eye) for 8 h. PBS-injected eyes were used as a control. Infected and control eyes were subjected to RNA isolation and qRT-PCR for *Tnfα*, *Il-1β*, and *Il-6* cytokine genes. Data represent mean ± standard error of the mean (SEM); *n* = 3; ns, not significant; ** *p* < 0.01; *** *p* < 0.001; Student’s *t*-test.

Inflammatory mediators are known to recruit innate immune cells to the site of injury or infection. To test this possibility, we used quantitative real-time PCR and assessed the expression of key inflammatory mediators in zebrafish eyes following bacterial infection. Our data showed that *S. aureus*-infected zebrafish eyes expressed significantly higher levels of transcripts of both *Il-1β* and *Il-6* inflammatory cytokines, whereas levels of *Tnf-α* were similar to uninfected control eyes (Figure 6). These results demonstrated the induction of the innate inflammatory response in the zebrafish eyes following *S. aureus* challenge.

## 3. Discussion

While zebrafish is increasingly being used as an infectious disease model, to our knowledge, its susceptibility to ocular infection has not been investigated. In this study, we explored the possibility of developing a zebrafish model of bacterial endophthalmitis to provide new insights into the pathogenesis of this blinding eye disease. We report that regardless of infectious dose, zebrafish eyes were able to rapidly clear the infection (*S. aureus*), resulting in no ocular/retinal tissue damage, which is typically seen in human clinical findings and a murine model of this disease. Moreover, intraocular inoculated bacteria appeared to be cleared through the retinal vasculature and phagocytic activities of infiltrated monocytes/macrophages in the zebrafish eyes. Given the close resemblance between the human and zebrafish immune systems [23,24], a better understanding of the protective innate immune mechanisms operating in zebrafish could lead to the identification of new host targets. 

Among bacterial pathogens, *Staphylococci* remain the leading cause of bacterial endophthalmitis, with *S. aureus* infection resulting in severe disease outcomes and often leading to visual disability and blindness [27]. In clinical settings, most bacterial endophthalmitis arises due to postsurgical complications (e.g., cataract surgery), where pathogens from the ocular surface gain access to the eye and cause ocular tissue damage [28,29]. To mimic this situation, studies from our [7,26,30] and other laboratories [31,32] have developed murine models where bacteria are directly inoculated in the vitreous cavity. Previous studies have demonstrated that *S. aureus* caused endophthalmitis in mice when eyes were inoculated with 5000 or less CFUs [7,10,33,34]. In contrast to the mice, we found that zebrafish eyes infected with 5000 CFU of *S. aureus* did not cause any ocular pathology. Therefore, we postulated that zebrafish might need a higher inoculum to cause endophthalmitis. Surprisingly, our dose–response study revealed that even the intravitreal injection of a 50-fold higher dose, i.e., 250,000 CFU/eye, did not cause pathology in the zebrafish eyes, as was evident by their intact retinal architecture. These findings indicate that zebrafish are resistant to *S. aureus* endophthalmitis. 

Being an immune-privileged organ, the eye is conducive to the proliferation of various endophthalmitis causing bacteria. Moreover, there was a time-dependent increase in bacterial growth in the infected mouse eyes. Our bacterial burden analysis revealed that after inoculation, bacterial growth increased up to 8 h followed by a decline at 24 h; and by 48 and 72 h, bacteria were cleared from the eye. This data suggests that zebrafish eyes could mount an adequate immune response to clear the infection and maintain retinal integrity. To study the potential mechanisms of bacterial clearance, we utilized GFP-labeled *S. aureus*, as reported in our prior study [35]. We discovered that within 2 h post-bacterial infection, GFP-positive *S. aureus* was localized in the choroid, and at the 8-h time point, bacteria was present both in the choroid and inside the retinal blood vessels. Similarly to the bacterial plate count assay, this histological analysis revealed that bacteria were gradually cleared from the eyes within 48–72 h, as evidenced by the lack of GFP positivity. Moreover, we observed that *S. aureus* within the vitreous colocalized with innate immune cells, primarily monocyte/macrophages. Collectively, these results indicate that bacteria were cleared from the zebrafish eyes via the combined action of retinal blood vessels and the phagocytic activities of infiltrated innate immune cells in the vitreous. 

The presence of monocyte/macrophages in the infected zebrafish eyes indicated the induction of the innate immune response. In mouse eyes, polymorphonuclear neutrophils (PMN) are the prominent immune cells infiltrated during endophthalmitis to contain bacterial proliferation. The depletion of PMNs has been shown to increase the bacterial burden in the eye [7,30]. Since the recruitment and activation of innate immune cells are regulated by the production of inflammatory mediators, [7,11,36], we assessed their expression in infected zebrafish eyes. Our data showed a significantly increased expression of *Il-1β* and *Il-6,* and no changes were observed in the levels of *Tnf-α* in the control versus the *S. aureus*-infected eyes. While the production of inflammatory mediators is a protective host response during infection, their excessive levels could lead to collateral tissue damage [29]. Among the various inflammatory mediators produced, *Tnf-α* has been shown to cause retinal tissue damage in several ocular diseases [37] and has been reported to exert a protective role in *Bacillus* endophthalmitis [38]. Similarly, *Il-1β* has been shown to protect the host from *S. aureus* infection in various models [39,40]. Further studies are needed to dissect the role of these individual cytokines in protecting zebrafish eyes from *Staphylococcal* endophthalmitis.

Zebrafish have been used as an attractive alternative model to study host–pathogen interactions and innate immunity due to several advantages, including cost-effectiveness, rapid breeding, and their close resemblance to the human immune system [23,24]. In conclusion, our study demonstrates that zebrafish eyes are resistant to bacterial endophthalmitis even when challenged with a higher dose of *S. aureus* in comparison to mouse models. Zebrafish have a profound ability to clear the pathogen from eyes and protect retinal tissue integrity, thus maintaining normal vision. The ability of zebrafish to mount a protective innate response is mediated via bacterial phagocytic clearance by monocytes/macrophages. Further studies are needed to determine whether similar mechanisms operate in zebrafish eyes infected with other endophthalmitis-causing pathogens, including fungi. 

## 4. Materials and Methods

### 4.1. Animals and Bacterial Maintenance

Wild-type (WT) zebrafish (*Danio rerio*) (AB strain, 9–12 months old) were used for this study and kept in standard laboratory conditions using a light schedule of 14 h on and 10 h off at a temperature of 28.5 °C [41]. Fish were fed daily using a combination of dry food and brine shrimp. Following injections, zebrafish were not fed and were closely monitored for any adverse reactions to bacterial injections. C57BL/6 WT mice (6–8 weeks old) were purchased from Jackson Laboratory, maintained at the Department of Laboratory Animal Resources (DLAR) facility with a 12:12 light–dark cycle, and fed LabDiet rodent chow and water ad libitum. All animal care and experimental protocols used in this study were approved by the Institutional Animal Care and Use Committee at Wayne State University and were in compliance with the Association for Research in Vision and Ophthalmology (ARVO) statement on the use of animals in vision research.

GFP-expressing *Staphylococcus aureus* (AL1743) (harboring chloramphenicol resistance) were maintained in tryptic soy broth/agar containing chloramphenicol. Prior to injection, bacteria were cultured in tryptic soy broth with chloramphenicol (20 µg/mL) overnight at 37 °C adjusted to a dosage of 250,000 CFU/0.5 µl in PBS.

### 4.2. S. aureus Intraocular Injections

Zebrafish were anesthetized, and a small incision was made at the edge of the cornea (a third of the corneal diameter long) using a scalpel (Safety Sideport Straight Knife 15°; Beaver-Vistec International). *S. aureus* AL1743 suspension (250,000 CFU/0.5 µL/eye) was injected into the vitreous chamber through an incision into the eye using a blunt-end 33-gauge Hamilton syringe. Fish were then placed back into water and incubated various times. In C57BL/6 mice, endophthalmitis was induced by giving an intravitreal injection of *S. aureus* (5000 CFU/eye) as described previously [7].

### 4.3. Bacterial Burden Estimation

The bacterial burden from zebrafish eyes was estimated using serial dilution and the plate count method. At each respective time point, fish were euthanized using 0.4mg/L of 2-phenoxyethanol. The eyes were enucleated and homogenized in sterile PBS using a Dounce homogenizer. The homogenate was serially diluted in sterile PBS, plated on tryptic soy agar plates containing chloramphenicol (20 µg/mL), and incubated at 37 °C. Following growth, the bacterial colonies were counted, and the results are expressed as the mean number of CFU/eye ± standard deviation (SD).

### 4.4. Immunohistochemistry

Following euthanasia, the eyes were enucleated and fixed for 1 h in 4% paraformaldehyde solution at room temperature. Following a PBS wash for 30 min, the eyes were then cryoprotected using a sucrose gradient of 5%–20%. The eyes were embedded in Optimal Cutting Temperature (OCT) Medium and sectioned. The frozen sections were dried for 2 h at 50 °C, followed by rehydration in PBS. The cryosections were blocked using a blocking solution consisting of 2% normal goat serum, 1% dimethyl sulfoxide (DMSO), and 0.2% Triton-X 100 in PBS for 1 h at room temperature. The sections were then incubated with primary antibodies diluted in blocking solution overnight at 4 °C. The primary antibody used in this study was rabbit monoclonal anti-GFP (1:1000, Abcam). The sections were washed with PBS containing 0.05% Tween-20 (PBST) and incubated for 1 h with Alexa Flour 488-labeled secondary antibodies. Nuclei were stained using TO-PRO-3 (TP3; 1:750; Life Technologies, Grand Island, NY). Sections were washed with PBST and mounted with ProLong Gold mounting media (Molecular Probes, Eugene, OR, USA). The sections were observed and imaged using a Leica TCS SP8 confocal microscope.

### 4.5. qRT-PCR

All RNA were isolated from zebrafish eyes using the Trizol method per the manufacturer’s protocol (Invitrogen), cDNA were prepared, and quantitative real-time PCR (qRT-PCR) was performed for inflammatory cytokines (*Tnfα*, *Il1β*, and *Il6*) using SYBR green-based primers on a CFX Connect Real-Time System (Bio-Rad). The quantification of gene expression was determined via the comparative ΔΔCT method. Gene expression in the test samples was normalized to the endogenous control, *gapdh*, and was reported as fold change relative to *gapdh* gene expression.

### 4.6. Statistical Analysis

All data are expressed as the mean ± standard error of the mean (SEM) unless indicated otherwise. Statistical differences between experimental groups were determined using Student’s *t*-test. All statistical analyses were performed using GraphPad Prism 8 (GraphPad Software, La Jolla, CA, USA). A value of *p* < 0.05 was considered statistically significant.

## References

[1] Callegan M.C., Gilmore M.S., Gregory M., Ramadan R.T., Wiskur B.J., Moyer A.L., Hunt J.J., Novosad B.D. (2007). Bacterial endophthalmitis: Therapeutic challenges and host-pathogen interactions. Prog. Retin. Eye Res..

[2] Taylor H. (2000). Cataract: How much surgery do we have to do?. Br. J. Ophthalmol..

[3] Chiquet C., Cornut P.L., Benito Y., Thuret G., Maurin M., Lafontaine P.O., Pechinot A., Palombi K., Lina G., Bron A. (2008). Eubacterial PCR for bacterial detection and identification in 100 acute postcataract surgery endophthalmitis. Investig. Ophthalmol. Vis. Sci..

[4] Campbell R.J., Bronskill S.E., Bell C.M., Paterson J.M., Whitehead M., Gill S.S. (2010). Rapid expansion of intravitreal drug injection procedures, 2000 to 2008: A population-based analysis. Arch. Ophthalmol..

[5] Sadaka A., Durand M.L., Gilmore M.S. (2012). Bacterial endophthalmitis in the age of outpatient intravitreal therapies and cataract surgeries: Host-microbe interactions in intraocular infection. Prog. Retin. Eye Res..

[6] Tosi M.F. (2005). Innate immune responses to infection. J. Allergy Clin. Immunol..

[7] Talreja D., Singh P.K., Kumar A. (2015). In Vivo Role of TLR2 and MyD88 Signaling in Eliciting Innate Immune Responses in Staphylococcal Endophthalmitis. Investig. Ophthalmol. Vis. Sci..

[8] Streilein J.W. (2003). Ocular immune privilege: Therapeutic opportunities from an experiment of nature. Nat. Rev. Immunol..

[9] Moyer A.L., Ramadan R.T., Novosad B.D., Astley R., Callegan M.C. (2009). Bacillus cereus-induced permeability of the blood-ocular barrier during experimental endophthalmitis. Investig. Ophthalmol. Vis. Sci..

[10] Callegan M.C., Booth M.C., Jett B.D., Gilmore M.S. (1999). Pathogenesis of gram-positive bacterial endophthalmitis. Infect. Immun..

[11] Kumar A., Kumar A. (2015). Role of Staphylococcus aureus Virulence Factors in Inducing Inflammation and Vascular Permeability in a Mouse Model of Bacterial Endophthalmitis. PLoS ONE.

[12] Singh P.K., Donovan D.M., Kumar A. (2014). Intravitreal Injection of the Chimeric Phage Endolysin Ply187 Protects Mice from Staphylococcus aureus Endophthalmitis. Antimicrob. Agents Chemother..

[13] Neely M.N., Pfeifer J.D., Caparon M. (2002). Streptococcus-zebrafish model of bacterial pathogenesis. Infect. Immun..

[14] Mitchell K.C., Breen P., Britton S., Neely M.N., Withey J.H. (2017). Quantifying Vibrio cholerae Enterotoxicity in a Zebrafish Infection Model. Appl. Environ. Microbiol..

[15] Runft D.L., Mitchell K.C., Abuaita B.H., Allen J.P., Bajer S., Ginsburg K., Neely M.N., Withey J.H. (2014). Zebrafish as a natural host model for Vibrio cholerae colonization and transmission. Appl. Environ. Microbiol..

[16] Howlader D.R., Sinha R., Nag D., Majumder N., Mukherjee P., Bhaumik U., Maiti S., Withey J.H., Koley H. (2016). Zebrafish as a novel model for non-typhoidal Salmonella pathogenesis, transmission and vaccine efficacy. Vaccine.

[17] Hall C.J., Boyle R.H., Astin J.W., Flores M.V., Oehlers S.H., Sanderson L.E., Ellett F., Lieschke G.J., Crosier K.E., Crosier P.S. (2013). Immunoresponsive gene 1 augments bactericidal activity of macrophage-lineage cells by regulating beta-oxidation-dependent mitochondrial ROS production. Cell Metab..

[18] Li Y., Li Y., Cao X., Jin X., Jin T. (2017). Pattern recognition receptors in zebrafish provide functional and evolutionary insight into innate immune signaling pathways. Cell. Mol. Immunol..

[19] Oosterhof N., Boddeke E., van Ham T.J. (2015). Immune cell dynamics in the CNS: Learning from the zebrafish. Glia.

[20] Sullivan C., Kim C.H. (2008). Zebrafish as a model for infectious disease and immune function. Fish Shellfish Immunol..

[21] Tobin D.M., May R.C., Wheeler R.T. (2012). Zebrafish: A see-through host and a fluorescent toolbox to probe host-pathogen interaction. PLoS Pathog..

[22] Zhu Z., Chen J., Xiong J.W., Peng J. (2014). Haploinsufficiency of Def activates p53-dependent TGFbeta signalling and causes scar formation after partial hepatectomy. PLoS ONE.

[23] Trede N.S., Zapata A., Zon L.I. (2001). Fishing for lymphoid genes. Trends Immunol..

[24] Postlethwait J.H., Yan Y.L., Gates M.A., Horne S., Amores A., Brownlie A., Donovan A., Egan E.S., Force A., Gong Z. (1998). Vertebrate genome evolution and the zebrafish gene map. Nat. Genet..

[25] Rajamani D., Singh P.K., Rottmann B.G., Singh N., Bhasin M.K., Kumar A. (2016). Temporal retinal transcriptome and systems biology analysis identifies key pathways and hub genes in Staphylococcus aureus endophthalmitis. Sci. Rep..

[26] Kumar A., Giri S., Kumar A. (2016). 5-Aminoimidazole-4-carboxamide ribonucleoside-mediated adenosine monophosphate-activated protein kinase activation induces protective innate responses in bacterial endophthalmitis. Cell. Microbiol..

[27] Gregory M., Callegan M.C., Gilmore M.S. (2007). Role of bacterial and host factors in infectious endophthalmitis. Chem. Immunol. Allergy.

[28] Kumar A., Pandey R.K., Miller L.J., Singh P.K., Kanwar M. (2013). Muller glia in retinal innate immunity: A perspective on their roles in endophthalmitis. Crit. Rev. Immunol..

[29] Miller F.C., Coburn P.S., Huzzatul M.M., LaGrow A.L., Livingston E., Callegan M.C. (2019). Targets of immunomodulation in bacterial endophthalmitis. Prog. Retin. Eye Res..

[30] Talreja D., Kaye K.S., Yu F.S., Walia S.K., Kumar A. (2014). Pathogenicity of ocular isolates of Acinetobacter baumannii in a mouse model of bacterial endophthalmitis. Investig. Ophthalmol. Vis. Sci..

[31] Parkunan S.M., Randall C.B., Coburn P.S., Astley R.A., Staats R.L., Callegan M.C. (2015). Unexpected Roles for Toll-Like Receptor 4 and TRIF in Intraocular Infection with Gram-Positive Bacteria. Infect. Immun..

[32] Hunt J.J., Astley R., Wheatley N., Wang J.-T., Callegan M.C. (2014). TLR4 Contributes to the Host Response to Klebsiella Intraocular Infection. Curr. Eye Res..

[33] Whiston E.A., Sugi N., Kamradt M.C., Sack C., Heimer S.R., Engelbert M., Wawrousek E.F., Gilmore M.S., Ksander B.R., Gregory M.S. (2008). alphaB-crystallin protects retinal tissue during Staphylococcus aureus-induced endophthalmitis. Infect. Immun..

[34] Engelbert M., Gilmore M.S. (2005). Fas ligand but not complement is critical for control of experimental Staphylococcus aureus Endophthalmitis. Investig. Ophthalmol. Vis. Sci..

[35] Kochan T., Singla A., Tosi J., Kumar A. (2012). Toll-Like Receptor 2 Ligand Pretreatment Attenuates Retinal Microglial Inflammatory Response but Enhances Phagocytic Activity toward Staphylococcus aureus. Infect. Immun..

[36] Giese M.J., Sumner H.L., Berliner J.A., Mondino B.J. (1998). Cytokine expression in a rat model of Staphylococcus aureus endophthalmitis. Investig. Ophthalmol. Vis. Sci..

[37] Nakazawa T., Kayama M., Ryu M., Kunikata H., Watanabe R., Yasuda M., Kinugawa J., Vavvas D., Miller J.W. (2011). Tumor Necrosis Factor-α Mediates Photoreceptor Death in a Rodent Model of Retinal Detachment. Investig. Ophthalmol. Vis. Sci..

[38] Ramadan R.T., Moyer A.L., Callegan M.C. (2008). A role for tumor necrosis factor-alpha in experimental Bacillus cereus endophthalmitis pathogenesis. Investig. Ophthalmol. Vis. Sci..

[39] Pires S., Parker D. (2018). IL-1β activation in response to Staphylococcus aureus lung infection requires inflammasome-dependent and independent mechanisms. Eur. J. Immunol..

[40] Miller L.S., Pietras E.M., Uricchio L.H., Hirano K., Rao S., Lin H., O’Connell R.M., Iwakura Y., Cheung A.L., Cheng G. (2007). Inflammasome-Mediated Production of IL-1β Is Required for Neutrophil Recruitment against *Staphylococcus aureus* In Vivo. J. Immunol..

[41] Westerfield M. (2000). The Zebrafish Book. A Guide for the Laboratory Use of Zebrafish (Danio Rerio).

